# A role of ^18^F-fluorodeoxyglucose positron emission/computed tomography in a strategy for abdominal wall metastasis of colorectal mucinous adenocarcinoma developed after laparoscopic surgery

**DOI:** 10.1186/1477-7819-9-28

**Published:** 2011-02-28

**Authors:** Kimihiko Funahashi, Mitsunori Ushigome, Hironori Kaneko

**Affiliations:** 1 Department of Gastroenterological Surgery, Toho University Medical Center, Omori Hospital, 6-11-1 Omori nishi, Otaku, Tokyo, 143-8541, Japan

## Abstract

Metastasis to the abdominal wall including port sites after laparoscopic surgery for colorectal cancer is rare. Resection of metastatic lesions may lead to greater survival benefit if the abdominal wall metastasis is the only manifestation of recurrent disease. A 57-year-old man, who underwent laparoscopic surgery for advanced mucinous adenocarcinoma of the cecum 6 years prior, developed a nodule in the surgical wound at the lower right abdomen. Although tumor markers were within normal limits, the metastasis to the abdominal wall and abdominal cavity from the previous cecal cancer was suspected. An abdominal computed tomography scan did not provide detective evidence of metastasis. ^18^F-fluorodeoxyglucose positron emission/computed tomography (^18^F-FDG PET/CT) was therefore performed, which demonstrated increased ^18^F-fluorodeoxyglucose uptake (maximum standardized uptake value: 3.1) in the small abdominal wall nodule alone. Histopathological examination of the resected nodule confirmed the diagnosis of metastatic mucinous adenocarcinoma. Prognosis of intestinal mucinous adenocarcinoma is reported to be poorer than that of non-mucinous adenocarcinoma. In conclusion, this case suggests an important role of ^18^F-FDG PET/CT in early diagnosis and decision-making regarding therapy for recurrent disease in cases where a firm diagnosis of recurrent colorectal cancer is difficult to make.

## Background

Metastasis to the abdominal wall including port sites after laparoscopic surgery for colorectal carcinoma (CRC) is rare. Recently the rate was reported as 1.3% in a randomized clinical trial by the Colon Cancer Laparoscopic or Open Resection Study Group [1] and 2.4% in the CLASSIC trial [2]. Although the prognosis is not clearly defined in the literature, resection of metastatic lesions may lead to greater survival benefit if the abdominal wall metastasis is the only manifestation of recurrent disease. However, it can be difficult to diagnose a lesion in the abdominal wall as recurrence of disease on the basis of clinical characteristics alone. Approximately between 5% to 15% of CRCs are mucinous adenocarcinomas [3-7]. Patients with colorectal mucinous adenocarcinoma are reported to have a poorer prognosis compared to patients with non-mucinous adenocarcinoma because the greater frequency of lymph node involvement and peritoneal dissemination seen with mucinous adenocarcinoma [7-10]. Therefore, Patients with mucinous adenocarcinoma should be followed carefully after surgery, and receive rapid diagnosis and treatment if recurrence is suspected. We report a case in which ^18^F-fluorodeoxyglucose positron emission/computed tomography (^18^F-FDG PET/CT) was very useful for early diagnosis and planning a theraupetic strategy for a case of mucinous adenocarcinoma metastasis at a laparoscopic port site.

## Case presentation

**Figure 1:**
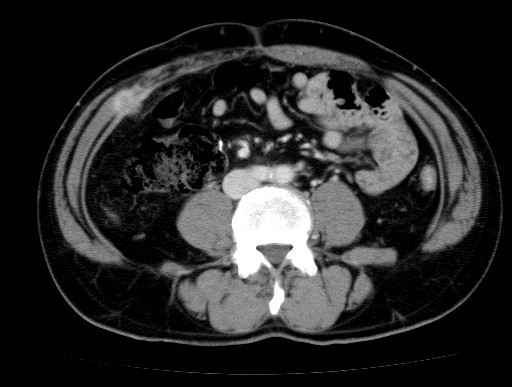
**Abdominal computed tomography scan**. Abdominal computed tomography scan on November 2008 revealed a small nodule in the abdominal wall, which was difficult to interpret as metastasis of cecal cancer by only computed tomography image.

**Figure 2:**
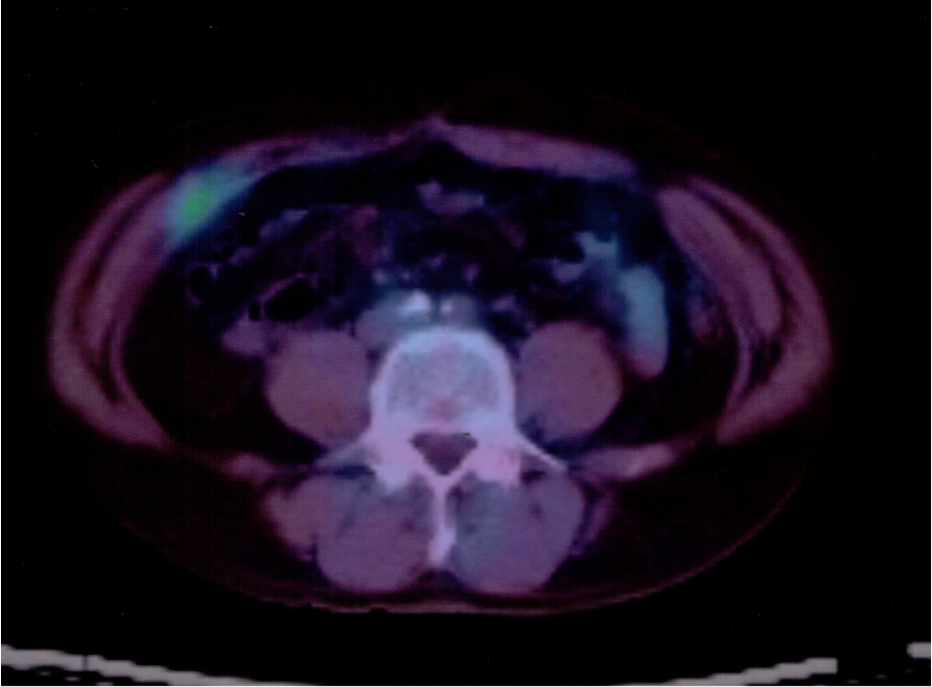
^**18**^**F-fluorodeoxyglucose positron emission/computed tomography**. ^18^F-fluorodeoxyglucose positron emission/computed tomography demonstrated increased ^18^F-fluorodeoxyglucose uptake (maximum standardized uptake value: 3.1) in the small nodule in the abdominal wall.

**Figure 3:**
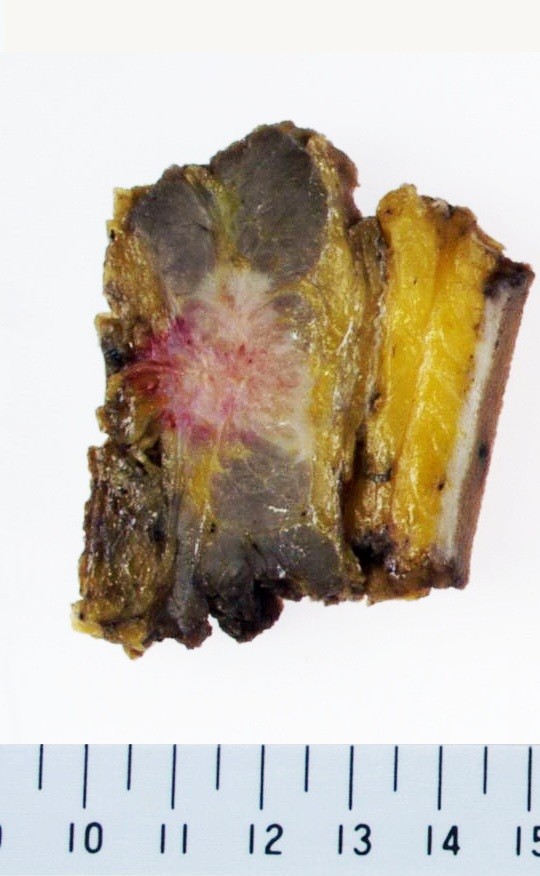
**Resected specimen**.

**Figure 4:**
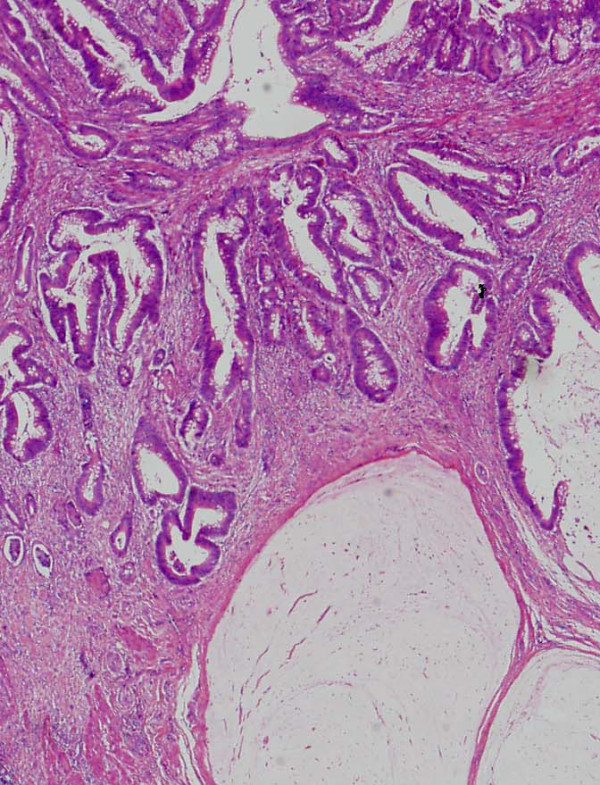
**Pathological findings**. Primary tumor. The histological examination revealed mucinous adenocarcinoma invading into the subserosa. Tumor cells in the regional lymph nodes, surgical margins, lymph vessels and veins were not identified histologically in the specimen (pT3 N0 M0).

**Figure 5:**
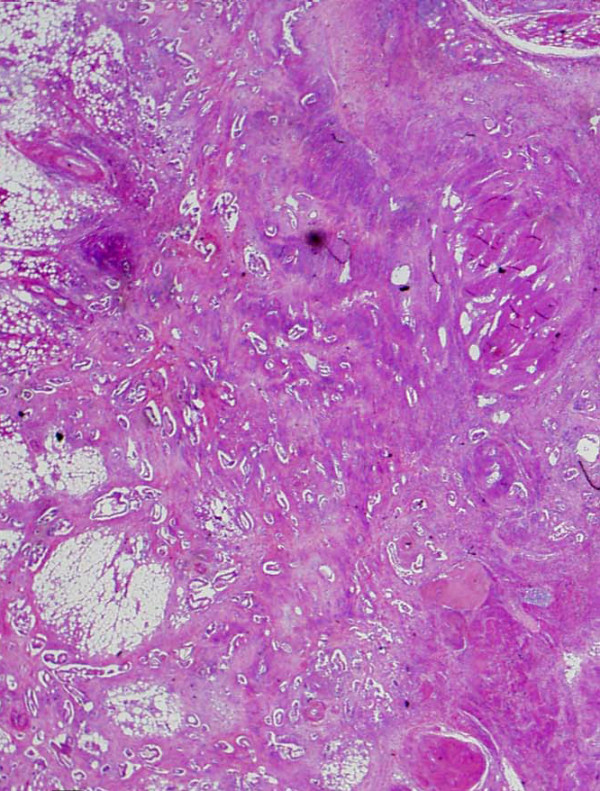
**Pathological findings**. Metastatic tumor. The tumor was located in the abdominal wall, slightly exposed to the abdominal cavity. Clinico-pathological findings showed the tumor was identified as a metastasis from cecal carcinoma.

A 57-year-old man received curative laparoscopic ileocecal resection and lymph node dissection for carcinoma of the cecum in May 2004. Morphologically, the tumor was type I (45 mm by 30 mm). The histological examination revealed a mucinous adenocarcinoma which invaded the cecal subserosa. Tumor cells were not identified histologically in the 20 regional lymph nodes, surgical margins, lymph vessels, or veins of the surgical specimens (pT3 N0 M0). The patient was subsequently followed at our hospital and treated with oral 5-fluorouracil. In February 2008, the patient discovered a nodule in the incision site in the lower right abdomen. A 2-cm, firm, ill-defined, tender mass was palpable in the incision site, and was suspected to be a recurrence of the cecal mucinous adenocarcinoma. However, the levels of carcinoembryonic antigen (CEA) and carbohydrate antigen 19-9 (CA19-9) were within normal limits (CEA: 4.7 ng/dl, CA19-9: 16.2 U/ml). In November 2008, an abdominal computed tomography (CT) scan revealed a small nodule in the abdominal wall, which was difficult to interpret as metastasis of the cecal cancer (Figure 1). 18F-fluorodeoxyglucose (^18^F-FDG) positron emission/computed tomography (PET/CT) was performed in January 2009. The CT scan was performed first, from head to pelvic floor using 3.3-mm section thickness. Immediately after the CT scan, a PET scan was performed using the identical transverse field of view and section thickness as that of the CT scan. For the PET scan, the patient, whose blood glucose level was 103 mg/dl, received 181.8 MBq of ^18^F-FDG intraverously. Data acquisition was performed within 20 min after injection using an integrated PET/CT system (Eminence SOPHIA; Shimadzu Corporation, Kyoto, Japan). PET image data sets were reconstructed by ^137^caesium for attenuation correction, and coregistered images were displayed. The PET/CT scan demonstrated increased ^18^F-FDG uptake (maximum standardized uptake value: 3.1) in the small abdominal nodule, but no further metastases in distant organs, peritoneum, or lymph nodes. The small nodule was diagnosed as a solitary metastasis of the cecal cancer at the previous port site (Figure 2). The nodule was resected in February 2009. The tumor was located in the abdominal wall, slightly exposed to the abdominal cavity. There was no gross evidence of metastasis in the abdominal cavity and cytological examination identified no tumor cells in the ascitic fluid. The tumor was identified as a metastatic lesion on the basis of histological findings (Figures 3, 4 and 5). No recurrence developed during 24-months postsurgical follow up.

## Discussion

Port site metastasis after laparoscopic surgery for CRC is rare, reported as 0.71-1% in the literature [11-16]. Recently the rate was reported as 1.3% in a randomized clinical trial by the Colon Cancer Laparoscopic or Open Resection Study Group [1] and 2.4% in the CLASSIC trial [2]. Several factors that may contribute to abdominal wall metastasis have been proposed [17], but it was impossible to identify a cause in this case. The operating record indicated that a wound drape had been used to prevent the implantation of tumor cells during surgery; clinico-pathologically, the depth of invasion of the primary tumor was confined to the intestinal wall and no vascular invasion was identified, and there were no postoperative complications.

Early resection of the metastatic lesion may lead to greater survival benefit, but early confirmation of metastatic disease on the basis of clinical characteristics alone is challenging. ^18^F-FDG PET/CT imaging, which both structural and functional information provide, is used to identify and stage various types of tumors because of its superiority to traditional imaging for diagnosing recurrent disease. In a retrospective comparison of PET versus PET/CT for the detection of CRC recurrence, the sensitivity, specificity and overall accuracy of PET were 80%, 69% and 75% respectively, compared with 89%, 92% and 90%, respectively, for PET/CT [18]. Goshen et al [19] reported ^18^F-FDG PET/CT was a sensitive tool for the diagnosis of 16 abdominal wall lesions in 12 CRC patients, who had moderately or well-differentiated adenocarcinoma. Kozugi et al reported that ^18^F-FDG PET was an important tool for the detection of port site recurrence of colon cancer in a patient who had elevated serum CEA levels but no metastases detected using routine radiographic examinations [20]. In addition, Sarikaya et al retrospectively analyzed the usefulness of PET for patients with CRC and suspected tumor recurrence, but normal CEA levels, and found that the overall accuracy of PET was 76.9%, and the positive predictive value was 84.6%. They concluded that PET yielded high positive predictive value for recurrence CRC despite normal CEA levels, and should be considered early in the evaluation of patients with suspected tumor recurrence [21]. ^18^F-FDG PET/CT is useful tool to help interpret potential malignancies when routine radiographic examinations are inconclusive. In addition, we consider that ^18^F-FDG PET/CT should be a prerequisite examination in patients with suspected recurrence of CRC who have normal CEA levels.

^18^F-FDG PET/CT imaging, however, does have some disadvantages. False-negative findings can occur for several reasons, including inflammation, small lesions size and diabetes. Mucinous adenocarcinoma as a histological type, regardless of the organs, may result in more false negatives as well. Sarikaya et al [21] reported that 3 of 5 patients (60%) with false-negative PET findings had mucinous adenocarcinoma diagnosed histologically. Rodriguez-Fernandez et al [22] and Sun L et al [23] reported false-negative results in patients with mucinous adenocarcinoma of the gallbladder and gastric cancer, respectively. For detection of gallbladder recurrence ^18^F-FDG PET scan showed a sensitivity of 80%, a specificity of 82%, and positive and negative predictive values of 67% and 90%, respectively. The single false-negative result was a patient with mucinous adenocarcinoma. For detection of gastric cancer recurrence, the accuracy of ^18^F-FDG PET/CT scan was 82.6%, and positive and negative predictive values were 85.7% and 77.7%, respectively. The two false-negative in patients with mucinous adenocarcinoma as shown in these reports, and in our case study it can be difficult to detect lesions of mucinous adenocarcinoma by PET scan and ^18^F-FDGPET/CT scan can be very useful in early diagnosis and therapeutic management.

Mucinous adenocarcinomas have a biological behavior that involves more lymph nodes at diagnosis and the greater frequency of peritoneal dissemination when compared to non-mucinous adenocarcinomas [7-10]. Recently, treatment with FOLFOX (Folinic acid + Fluorouracil + Oxaliplatin) or FOLFIRI (Folinic acid + Fluorouracil + Irinotecan) has been considered useful to obtain better progression-free survival for unresectable colorectal recurrence. However, there is no doubt that early complete resection of the metastatic lesion could lead to even greater survival benefit. ^18^F-FDG PET/CT scan can play an important role in selecting among patients with recurrence those who may obtain greater survival benefit.

## Conclusion

In the case we presented ^18^F-FDG PET/CT scan was very useful in early diagnosis and therapeutic management for recurrence of mucinous adenocarcinoma after laparoscopic surgery for CRC. Mucinous adenocarcinomas may contribute to a higher rate of false-negative results, but does not decrease the usefulness of this diagnostic tool. ^18^F-FDG PET/CT imaging, which provide both functional and anatomical information and correctly stages recurrence disease should be considered early in the evaluation of patients with suspected recurrence of CRC.

## Consent

Written informed consent was obtained from the patient for publication of this case report and any accompanying image. A copy of the written consent is available for review by the Editor-in Chief of this journal.

## Abbreviations

CRC: colorectal carcinoma; ^18^F-FDG PET/CT: ^18^F-fluorodeoxyglucose positron emission/computed tomography; ^18^F-FDG: ^18^F-fluorodeoxyglucose; PET/CT: positron emission/computed tomography; CEA: carcinoembryonic antigen; CA19-9: carbohydrate antigen 19-9; CT: computed tomography; PET: positron emission tomography; FOLFOX: Folinic acid + Fluorouracil + Oxaliplatin; FOLFIRI: Folinic acid + Fluorouracil + Irinotecan;

## Competing interests

The authors declare that they have no competing interests.

## Authors' contributions

MU was an assistant of the operation. HK is a chairman of the department of gastroenterological surgery, Toho University Medical Center, Omori Hospital. All authors read and approved the final manuscript.
